# ^18^F‐FDG PET/CT findings of paratesticular alveolar rhabdomyosarcoma

**DOI:** 10.1002/cai2.121

**Published:** 2024-04-08

**Authors:** Xueqi Chen, Jiayin Shou, Shanshi Li, Yan Fan, Jianhua Zhang

**Affiliations:** ^1^ Department of Nuclear Medicine Peking University First Hospital Beijing China; ^2^ Department of Radiation Oncology Peking University First Hospital Beijing China

**Keywords:** ^18^F‐FDG, paratesticular alveolar rhabdomyosarcoma, PET/CT

## Abstract

Rhabdomyosarcoma (RMS) originates from primitive mesenchymal cells and is the most common soft tissue tumor in childhood. ^18^F‐fluoro‐deoxyglucose (^18^F‐FDG) positron emission tomography (PET)/computed tomography (CT) has been reported to be valuable in RMS staging and risk stratification. Paratesticular RMS is a relatively uncommon form of RMS, most of which are of the embryonal histologic type. Paratesticular alveolar RMS is associated with aggressive behavior, high metastatic potential, and poor outcomes. To the best of our knowledge, ^18^F‐FDG PET/CT imaging findings of paratesticular alveolar RMS have never been described. Here, we report on a 16‐year‐old boy's rare paratesticular alveolar RMS with multiple metastases and its findings on ^18^F‐FDG PET/CT. This case also demonstrates the potential value of ^18^F‐FDG PET/CT in RMS staging and treatment decisions, and may aid in the differential diagnosis.

## Graphical abstract

We reported a rare case of paratesticular alveolar rhabdomyosarcoma (RMS) with multiple metastases in a 16‐year‐old boy and its findings on ^18^F‐fluoro‐deoxyglucose (^18^F‐FDG) positron emission tomography (PET)/computed tomography (CT). This case indicates the potential value of ^18^F‐FDG PET/CT in rhabdomyosarcoma staging and therapy decisions and may assist in the differential diagnosis.

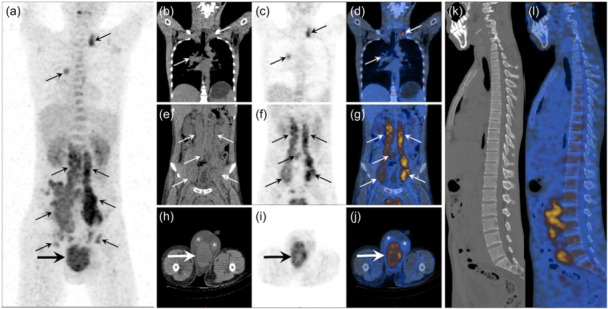

**Abbreviations**
^18^F‐FDG: ^18^F‐fluoro‐deoxyglucose
PET/CT: positron emission tomography/computed tomography
RMS: rhabdomyosarcoma

## 1 BACKGROUND

Rhabdomyosarcoma (RMS) originates from primitive mesenchymal cells, and is the most common soft tissue tumor in childhood, accounting for approximately half of all soft tissue sarcomas in this age group [1]. According to the World Health Organization (WHO) 2020 classification of soft tissue and bone tumors, RMS can be classified into four main histologic types: embryonal, alveolar, pleomorphic, and spindle/sclerosis subtypes. RMS occurs primarily in the head and neck, genitourinary tract, and extremities [1]. Paratesticular RMS develops from the spermatic cord, epididymis, and testicular capsule and accounts for 7% to 10% of cases of genitourinary RMS (23% of all RMS). Among them, the most common pathological subtype is embryonal variation (approximately 80%) [2]. Paratesticular RMS has a bimodal age distribution, peaking at 1 to 5 years of age and then at 16 years [3]. Treatment of paratesticular alveolar RMS includes surgery, multiagent chemotherapy, and radiation therapy [2]. Paratesticular alveolar RMS is rare and may be associated with aggressive behavior, high metastatic potential, and poor prognosis [4]. Here, we present a rare case of paratesticular alveolar RMS with distant metastases and its findings on ^18^F‐fluoro‐deoxyglucose (^18^F‐FDG) positron emission tomography (PET)/computed tomography (CT).

## 2 CASE STUDY

A 16‐year‐old boy presented with a 1‐month of history of painless scrotal swelling. Ultrasonography revealed a solid paratesticular mass and multiple enlarged inguinal lymph nodes. Laboratory tests showed elevated serum creatinine (809.3 µmol/L, reference range 44–133 µmol/L), uric acid (1117 µmol/L, reference range 90–360 µmol/L), LDH (1691 IU/L, reference range 100–240 IU/L), and normal alpha‐fetoprotein and beta‐human chorionic gonadotropin levels.

**Figure 1:**
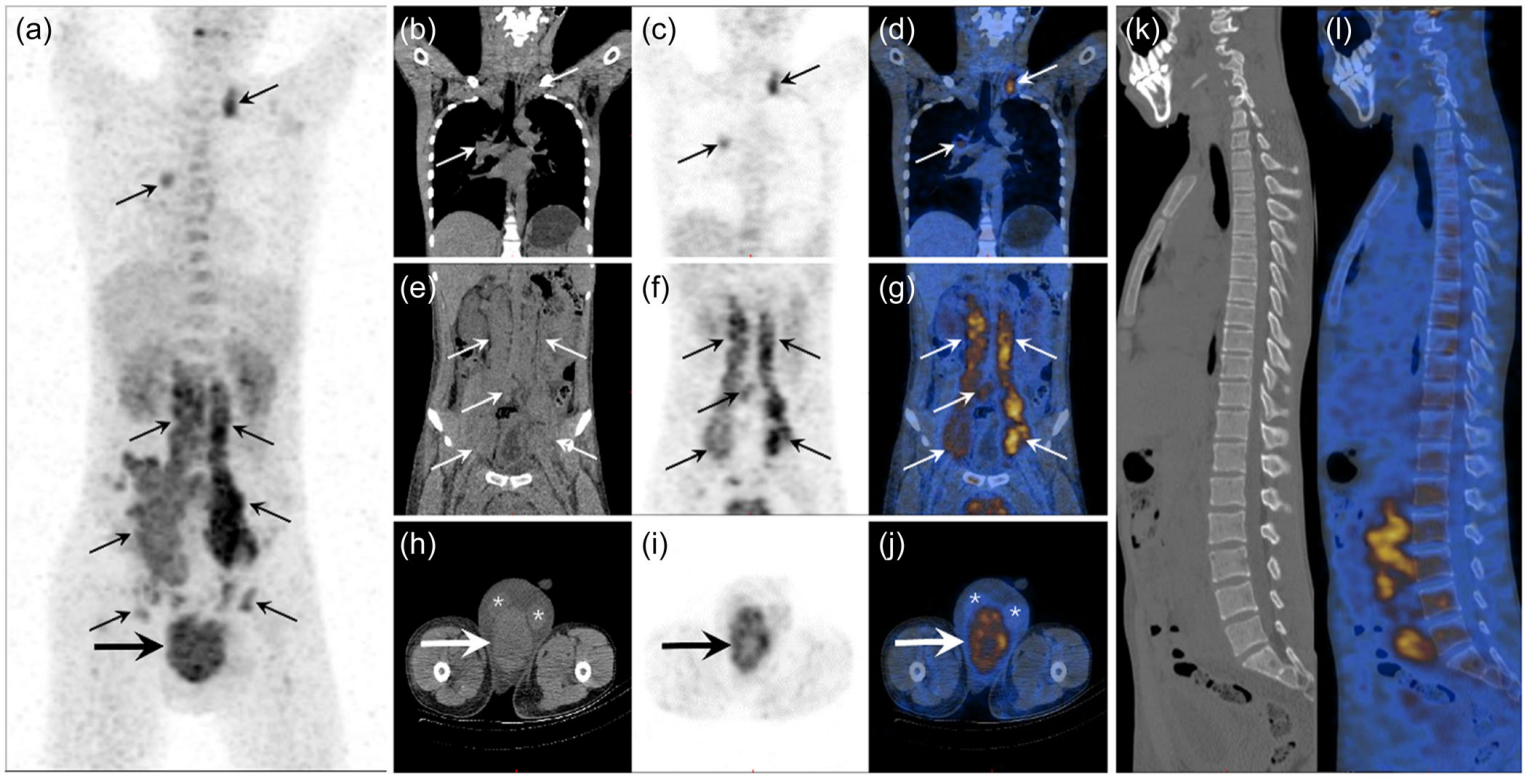
^18^F‐fluoro‐deoxyglucose (^18^F‐FDG) positron emission tomography (PET) /computed tomography (CT) images. (a) Maximal intensity projection (MIP) image. Strong uptake of ^18^F‐FDG in the scrotum (thick arrow), multiple lymph nodes (thin arrows) in left supraclavicular area, right hilum, abdomen, and bilateral inguinal region. (b–d) ^18^F‐FDG accumulation in the lymph nodes in left supraclavicular and right hilum (thin arrows) shown on coronal CT (b), PET (c), and fusion PET/CT (d) images. (e–g) ^18^F‐FDG accumulation in the retroperitoneal region (thin arrows) shown on coronal CT (e), PET (f), and fusion PET/CT (g) images. (h–j) Axial CT (h), PET (i), and fusion PET/CT (j) images of the paratesticular soft tissue mass located posterior to the bilateral testis (*) (measured 12.2 cm × 9.0 cm × 8.4 cm) (thick arrows) with well‐defined marginal boundaries and SUVmax of 6.0. (k, l) Multiple small scattered low‐density vertebral lesions (k) with heterogeneous ^18^F‐FDG accumulation (l).

An ^18^F‐FDG PET/CT was performed to investigate the paratesticular mass, distant metastases, or potential malignancy of disease extent. The maximal intensity projection (MIP) image showed a strong uptake of ^18^F‐FDG in the scrotum, multiple lymph nodes in the left supraclavicular area, right hilum, abdomen, and bilateral inguinal region. Slight heterogeneity was observed in multiple vertebral bodies with ^18^F‐FDG‐affinity. Coronal CT images, corresponding PET, and fused PET/CT revealed ^18^F‐FDG accumulation in the lymph nodes in the left supraclavicular, right hilum, and retroperitoneal region. The para‐aortic and iliac lymph nodes were severely enlarged and fused and showed the most active FDG affinity (SUVmax 7.5). On axial CT image, the paratesticular soft tissue mass (measured 12.2 cm × 9.0 cm × 8.4 cm) was posterior to the testis with a well‐defined marginal border. The corresponding PET and fusion PET/CT revealed increased ^18^F‐FDG activity with an SUVmax of 6.0 (Figure 1).

PET/CT findings suspected paratesticular malignancy with multiple metastases or extensive lymphoma involvement, while ultrasound‐guided core needle biopsy histopathologically showed paratesticular alveolar RMS and fluorescence in situ hybridization (FISH) showed positive *FOXO1* mutations. The clinical diagnosis of multiple lymph nodes and bone metastases was based on a comprehensive diagnostic evaluation that included PET/CT findings.

## 3 DISCUSSION

Alveolar RMS accounts for 15% of cases, tends to occur in older children, and has a worse prognosis compared with embryonal RMS. At the molecular level, most alveolar RMSs have a characteristic fusion of the *PAX3* or *PAX7* genes with *FOXO1*, which is caused by a specific chromosomal translocation [3]. In this setting, the presence of *FOXO1* rearrangements identified by FISH or reverse‐transcriptase polymerase chain reaction supports the diagnosis of alveolar RMS.

Imaging is important for the diagnosis and evaluation of RMS, including assessment of tumor spread, lymph node involvement, and distant metastases. Ultrasound, CT, and magnetic resonance imaging (MRI) have been widely used in the evaluation of RMS. Ultrasound can be used as an initial examination, particularly in the evaluation of scrotal masses. Patients typically present with a painless hypoechoic scrotal mass and a solid, heterogeneous extratesticular mass on ultrasound [2]. CT has advantages in detecting pulmonary nodules and assessing bone involvement [3, 5]. MRI is considered the best imaging modality for most primary RMS with moderate to high intensity on T1‐weighted and T2‐weighted images [6]. However, radiographic findings of RMS are largely nonspecific. ^18^F‐FDG PET/CT has been reported to be of great value in RMS staging and risk stratification, and is also used to detect unknown primary sites or sites of rare metastases, treatment decisions, prognostic prediction, and treatment response evaluation [7, 8].

Although the superficial location of paratesticular RMS facilitates early diagnosis, previous studies have shown that lymph node involvement is more common in patients with paratesticular RMS older than 10 years of age [5]. Therefore, these patients require special attention to lymph node evaluation. Although ^18^F‐FDG PET/CT may have advantages in identifying lymph node involvement compared with conventional imaging, with higher sensitivity for a single positive node and fewer uncertain results [9], ^18^F‐FDG PET/CT may present more closely to lymphoma in RMS [10]. Therefore, the accurate diagnosis of lymphatic metastases relies on biopsy core needle biopsy or resection rather than PET/CT [5]. In this case, ultrasound‐guided core needle biopsy results confirm paratesticular alveolar RMS and positive *FOXO1* mutations.

## 4 CONCLUSION

We present a rare case of paratesticular alveolar RMS with distant metastases, demonstrating the potential value of ^18^F‐FDG PET/CT in the staging and treatment decisions of RMS. Further exploration is warranted to validate the differential diagnostic value of malignancies with similar presentations and to explore the clinical prognostic potential of ^18^F‐FDG in larger studies.

## AUTHOR CONTRIBUTIONS

**Xueqi Chen**: Conceptualization (equal); data curation (lead); investigation (equal); methodology (equal); writing—original draft (equal). **Jiayin Shou**: Conceptualization (equal); investigation (equal); methodology (equal); writing—original draft (equal). **Shanshi Li**: Data curation (supporting); investigation (supporting); methodology (supporting); writing—original draft (supporting). **Yan Fan**: Conceptualization (equal); data curation (lead); funding acquisition (equal); investigation (equal); methodology (equal); supervision (equal); writing—review and editing (equal). **Jianhua Zhang**: Conceptualization (equal); data curation (lead); funding acquisition (equal); investigation (equal); methodology (equal); supervision (equal); writing—review and editing (equal).

## CONFLICT OF INTEREST STATEMENT

The authors declare no conflict of interest.

## ETHICS STATEMENT

Not applicable.

## INFORMED CONSENT

Written informed consent was obtained from the patient's next of kin for publication of this case study and accompanying images.

## Data Availability

The data that support the findings of this study are available from the corresponding author upon reasonable request.

## References

[1] Weiss AR , Lyden ER , Anderson JR , Hawkins DS , Spunt SL , Walterhouse DO , et al. Histologic and clinical characteristics can guide staging evaluations for children and adolescents with rhabdomyosarcoma: a report from the children's oncology group soft tissue sarcoma committee. J Clin Oncol. 2013;31(26):3226–3232. 10.1200/jco.2012.44.6476 23940218 PMC3757291

[2] Dangle PP , Correa A , Tennyson L , Gayed B , Reyes‐Múgica M , Ost M . Current management of paratesticular rhabdomyosarcoma. Urol Oncol. 2016;34(2):84–92. 10.1016/j.urolonc.2015.10.004 26572723

[3] Jawad N , McHugh K . The clinical and radiologic features of paediatric rhabdomyosarcoma. Pediatr Radiol. 2019;49(11):1516–1523. 10.1007/s00247-019-04386-5 31620851

[4] Jha P , Frölich AMJ , McCarville B , Navarro OM , Babyn P , Goldsby R , et al. Unusual association of alveolar rhabdomyosarcoma with pancreatic metastasis: emerging role of PET‐CT in tumor staging. Pediatr Radiol. 2010;40(8):1380–1386. 10.1007/s00247-010-1572-3 20180103 PMC2895865

[5] Rogers TN , Dasgupta R . Management of rhabdomyosarcoma in pediatric patients. Surg Oncol Clin N Am. 2021;30(2):339–353. 10.1016/j.soc.2020.11.003 33706904

[6] Mallinson PI , Chou H , Forster BB , Munk PL . Radiology of soft tissue tumors. Surg Oncol Clin N Am. 2014;23(4):911–936. 10.1016/j.soc.2014.06.006 25246054

[7] Huang D , Watal P , Drehner D , Dhar D , Chandra T . Rhabdomyosarcoma with diffuse bone marrow metastases. Cureus. 2022;14(2):e21863. 10.7759/cureus.21863 35265406 PMC8897967

[8] El‐Kholy E , El Nadi E , Hafez H , Ahmed S , Younes A , El‐Kenanii N , et al. Added predictive value of 18F‐FDG PET/CT for pediatric rhabdomyosarcoma. Nucl Med Commun. 2019;40(9):898–904. 10.1097/MNM.0000000000001040 31145205

[9] Federico SM , Spunt SL , Krasin MJ , Billup CA , Wu J , Shulkin B , et al. Comparison of PET‐CT and conventional imaging in staging pediatric rhabdomyosarcoma. Pediatr Blood Cancer. 2013;60(7):1128–1134. 10.1002/pbc.24430 23255260 PMC4266929

[10] Norman G , Fayter D , Lewis‐Light K , Chisholm J , McHugh K , Levine D , et al. An emerging evidence base for PET‐CT in the management of childhood rhabdomyosarcoma: systematic review. BMJ Open. 2015;5(1):e006030. 10.1136/bmjopen-2014-006030 PMC4289735 25573522

